# Homocysteine serum levels in patients with ruptured and unruptured intracranial aneurysms: a case-control study

**DOI:** 10.1055/s-0044-1779270

**Published:** 2024-02-07

**Authors:** João Paulo Mota Telles, Jefferson Rosi Junior, Vitor Nagai Yamaki, Nicollas Nunes Rabelo, Manoel Jacobsen Teixeira, Eberval Gadelha Figueiredo

**Affiliations:** 1 Universidade de São Paulo, Faculdade de Medicina, Departamento de Neurologia, São Paulo SP, Brazil.; 2 Universidade de São Paulo, Faculdade de Medicina, Divisão de Neurocirurgia, São Paulo SP, Brazil.

**Keywords:** Homocysteine, Intracranial Aneurysm, Subarachnoid Hemorrhage, Stroke, Homocisteína, Aneurisma Intracraniano, Hemorragia Subaracnóidea, Acidente Vascular Cerebral

## Abstract

**Background** There is very few data regarding homocysteine's influence on the formation and rupture of intracranial aneurysms.

**Objective** To compare homocysteine levels between patients with ruptured and unruptured intracranial aneurysms, and to evaluate possible influences of this molecule on vasospasm and functional outcomes.

**Methods** This is a retrospective, case-control study. We evaluated homocysteinemia differences between patients with ruptured and unruptured aneurysms; and the association of homocysteine levels with vasospasm and functional outcomes. Logistic regressions were performed.

**Results** A total of 348 participants were included: 114 (32.8%) with previous aneurysm rupture and 234 (67.2%) with unruptured aneurysms. Median homocysteine was 10.75µmol/L (IQR = 4.59) in patients with ruptured aneurysms and 11.5µmol/L (IQR = 5.84) in patients with unruptured aneurysms. No significant association was detected between homocysteine levels and rupture status (OR = 0.99, 95% CI = 0.96-1.04). Neither mild (>15µmol/L; OR = 1.25, 95% CI 0.32-4.12) nor moderate (>30µmol/L; OR = 1.0, 95% CI = 0.54-1.81) hyperhomocysteinemia demonstrated significant correlations with ruptured aneurysms. Neither univariate (OR = 0.86; 95% CI 0.71-1.0) nor multivariable age-adjusted (OR = 0.91; 95% CI = 0.75-1.05) models evidenced an association between homocysteine levels and vasospasm. Homocysteinemia did not influence excellent functional outcomes at 6 months (mRS≤1) (OR = 1.04; 95% CI = 0.94-1.16).

**Conclusion** There were no differences regarding homocysteinemia between patients with ruptured and unruptured intracranial aneurysms. In patients with ruptured aneurysms, homocysteinemia was not associated with vasospasm or functional outcomes.

## RESUMO

**Antecedentes** Existem poucos dados sobre a influência da homocisteína na formação e rotura de aneurismas intracranianos (AI).

**Objetivo** Comparar os níveis de homocisteína entre pacientes com AI rotos e não rotos e influências no vasoespasmo e resultados funcionais.

**Métodos** Estudo caso-controle, que avaliou as diferenças de homocisteinemia entre pacientes com aneurismas rotos e não rotos, além da associação entre níveis de homocisteína, vasoespasmo e estado funcional. Regressões logísticas foram realizadas.

**Resultados** Um total de 348 participantes foram incluídos: 114 (32,8%) com aneurismas rotos e 234 (67,2%) não rotos. A homocisteína mediana foi de 10,75µmol/L (IQR = 4,59) nos rotos e 11,5µmol/L (IQR = 5,84) nos não rotos. Não houve associação significativa entre os níveis de homocisteína e o status de ruptura (OR = 0,99, 95% CI = 0,96-1,04). Nem a hiperhomocisteinemia leve (>15µmol/L; OR = 1,25, 95% CI = 0,32-4,12) nem a moderada (>30µmol/L; OR = 1,0, 95% CI = 0,54-1,81) mostraram correlações significativas com aneurismas rotos. Modelos univariados (OR = 0,86; 95% CI = 0,71-1,0) e multivariados ajustados por idade (OR = 0,91; 95% CI = 0,75-1,05) não evidenciaram associação entre homocisteína e vasoespasmo. A homocisteinemia não influenciou resultados funcionais excelentes em seis meses (mRS ≤ 1) (OR = 1,04; 95% CI = 0,94-1,16).

**Conclusão** Não houve diferenças em relação à homocisteinemia entre pacientes com aneurismas intracranianos rotos e não rotos. Em pacientes com aneurismas rotos, a homocisteinemia não foi associada ao vasoespasmo ou resultados funcionais.

## INTRODUCTION

Homocysteine (homocysteine) is a non-essential amino acid involved in the methionine metabolism pathways. 1 Homocysteine has been implicated in several vascular pathologic processes since 1969, with the observation of children with homocystinuria who had atherosclerosis, myocardial infarction, stroke, and other atherothrombotic conditions at a very early age. 2

High levels of homocysteine are robustly associated with stroke. 3 4 5 Several meta-analyses have demonstrated an increased risk of ischemic stroke in patients with hyperhomocysteinemia, which motivated the scientific community to undertake randomized controlled trials of B12 and folate supplementation for stroke prevention. 5 6 7

There is very little data regarding homocysteine's influence on the formation and rupture of intracranial aneurysms, and the results are conflicting. 8 9 10 11 The objective of the present study is to compare serum levels of homocysteine between patients with ruptured and unruptured IA. Furthermore, we sought to evaluate the possible influences of this molecule on vasospasm and functional outcomes.

## METHODS

This is a retrospective, case-control methodology. An exhaustive review of medical records was conducted for all sequential patients who sought consultation at the cerebrovascular neurosurgery clinic of Hospital das Clínicas da Faculdade de Medicina da Universidade de Sao Paulo (HC-FMUSP). The research project received approval from the HC-FMUSP's ethics review board, consistent with the principles articulated in the Helsinki Declaration, and the necessity for informed consent was excused due to the retrospective nature of the study (approval number 5.576.011, 2022).

### Population

The study's inclusion criteria comprised patients diagnosed with intracranial aneurysms, irrespective of rupture history, who also had homocysteine serum levels measured. Exclusion criteria were set as patients below the age of 18, those with arteriovenous malformations or concurrent cerebrovascular diseases excluding prior strokes, or individuals in the acute post-stroke phase, defined as less than one-month post-discharge.

Demographic data were gathered, which incorporated variables such as age, gender, pre-existing health conditions, current smoking habits, a history of ischemic stroke, and statin usage. For patients with a history of ruptured aneurysms, inpatient records were meticulously examined to detect any incidence of vasospasm, documented on angiotomography, angiography, or transcranial Doppler. Neurological outcomes were subsequently evaluated utilizing the modified Rankin Scale (mRS). 12

Homocysteine levels were obtained in the outpatient environment, with a time frame ranging from a minimum of one month to a maximum of six months post-discharge. For patients with unruptured aneurysms, serum levels were obtained when first evaluated in the outpatient clinic before any treatments were considered. Serum homocysteine concentrations were considered as both a continuous and a dichotomous variable - hyperhomocysteinemia was classified as a homocysteine level ≥ 15 µmol/L (mild), homocysteine level ≥ 30 µmol/L (moderate), or homocysteine level > 100 µmol/L (severe). Since folate and vitamin B12 play important roles in the physiology of homocysteine, data on these vitamins were collected, to correct for potential confounding.

### Outcomes

The primary outcomes encompassed:

• disparities in serum homocysteine between patients with ruptured and unruptured aneurysms;
• the impact of homocysteine concentrations on functional status;
• the effect of homocysteine levels on vasospasm occurrence.

### Statistical analyses

Continuous data are depicted as either mean (±standard deviation) or median (interquartile range), subject to the results of normality assessments (Shapiro-Wilk's test). T-tests were employed for comparing normally distributed continuous variables, while non-parametric variables were compared using the Mann-Whitney U tests. Categorical variables are represented as frequencies (valid %) and underwent comparison using chi-squared tests.

To evaluate the effect of homocysteine on binary outcomes – aneurysm rupture, vasospasm, and excellent functional outcome (defined as mRS ≤ 1), logistic regression analyses were performed. Covariates were incorporated into multivariable analyses on the basis of imbalances in baseline characteristics and the plausibility of their biological impact. Interactions were tested and non-linear correlations were assessed in accordance with diagnostic graphs for the models, where appropriate. All analyses were executed using R (R Foundation for Statistical Computing, Vienna, Austria, 2021).

## RESULTS

### Population characteristics

The characteristics of the study population are summarized in Table 1 . This study included a total of 348 participants, of which 114 (32.8%) had experienced a previous aneurysm rupture and 234 (67.2%) had unruptured aneurysms. The average age of patients with ruptured aneurysms was 53.9 (±13.0) years, while those with unruptured aneurysms had a mean age of 58.6 (±12.0) years (P < 0.01). Females comprised 73.7% and 78.2% of the ruptured and unruptured patient groups, respectively (P = 0.42).

**Table 1:** Baseline characteristics

Characteristics	Ruptured (n =114)	Unruptured (n = 234)	P-value
Age	53.9 (±13.0)	58.6 (±12.0)	< 0.01
Female	84 (73.7%)	183 (78.2%)	0.42
Smoking	35 (30.7%)	71 (30.3%)	1.00
Hypertension	31 (27.2%)	81 (34.6%)	0.20
Previous ischemic stroke	0 (0%)	12 (5.1%)	0.03
Dyslipidemia	26 (22.8%)	63 (26.9%)	0.49
Statin use	4 (3.5%)	22 (9.4%)	0.08

Notes: Data presented as mean (±standard deviation) or count (valid percentage). P-values refer to Welch t-test or Chi-squared tests.

Current smoking was reported by 30.7% and 30.3% (P = 1.0), a diagnosis of hypertension was established in 27.2% and 34.6% (P = 0.2), dyslipidemia was identified in 22.8% and 26.9% (P = 0.49), and statin usage was noted in 3.5% and 9.4% (P = 0.08) of the patients with ruptured and unruptured aneurysms, correspondingly. A history of prior ischemic stroke was confirmed in 12 (5.1%) patients with unruptured aneurysms, with no recorded instances within the ruptured aneurysm group (P = 0.03). With the exception of age and the incidence of prior ischemic stroke, no significant discrepancies were observed between the groups in relation to the baseline characteristics.

### Ruptured versus unruptured aneurysms

The median homocysteine level was 10.75 µmol/L (IQR = 4.59) in the ruptured aneurysm group, and 11.5 µmol/L (IQR = 5.84) in the unruptured group (OR 0.98, 95% CI 0.94-1.02). Table 2 further delineates median levels of B12 and folate, both of which were scrutinized to account for potential confounding effects on homocysteine levels. Neither were found to be associated with aneurysm rupture: OR 1.0 (95% CI 0.99-1.01) for B12 and OR 0.99 (0.94-1.03) for folate.

**Table 2:** Homocysteine, B12 and folate comparison between ruptured and unruptured aneurysms: univariate

	Ruptured	Unruptured	OR	95% CI
**Homocysteine (µmol/L)**	10.75 (4.59)	11.5 (5.84)	0.98	0.94-1.02
**B12 (pmol/L)**	442.5 (198)	393.5 (211.5)	1.00	0.99-1.01
**Folate (ng/mL)**	11.5 (5.65)	11.5 (7.5)	0.99	0.94-1.03

Notes: Median (interquartile range) of homocysteine, B12 and folate serum levels. Odds ratios (OR) refer to univariate logistic regression models comparing serum levels of these molecules (continuous) between patients with ruptured and unruptured aneurysms.

Multivariable analyses, compensating for disparities between groups, were conducted ( Table 3 ). No significant association was detected between homocysteine levels and rupture status (OR 0.99, 95% CI 0.96-1.04), after adjustments for age, prior ischemic stroke, and statin use. Tested interaction terms between homocysteine and the covariates revealed no statistical significance.

**Table 3:** Influence of homocysteine continuous and dichotomized on rupture of intracranial aneurysms: multivariable analyses

		OR	95% CI
Continuous	Homocysteine*	0.99	0.96–1.04
Dichotomized	Hcy > 30 µmol/L**	1.25	0.32-4.12
	Hcy > 15 µmol/L**	1.00	0.54-1.81
Hcy, B12, and Folate on the same model	Homocysteine	0.98	0.93-1.02
	B12	1.00	0.99-1.01
	Folate	0.98	0.93-1.02

Abbreviations: CI, confidence interval; Hcy, homocysteine; OR, odds ratio.

Notes: *Adjusted for age, previous ischemic stroke, and statin use. Interaction terms were also non-significant; ** Age-adjusted.

When homocysteine was evaluated as a dichotomous variable and adjusted for age, neither mild (> 15 µmol/L; OR 1.25, 0.32-4.12) nor moderate (> 30 µmol/L; OR 1.0, 0.54-1.81) hyperhomocysteinemia demonstrated significant correlations with ruptured aneurysms. Lastly, a multivariable model incorporating homocysteine, B12, and folate was executed ( Table 3 ). None of these biochemical entities revealed any significant associations with ruptured aneurysms. Interactions terms were tested, yielding non-significant results as well.

### Vasospasm and functional outcomes

In total, fifteen patients were diagnosed with vasospasm, comprising 13.2% of the ruptured aneurysm group. Neither univariate (OR 0.86; 95% CI 0.71-1.0) nor multivariable age-adjusted (OR 0.91; 95% CI 0.75-1.05) models evidenced a significant association when assessing correlations between homocysteine levels and vasospasm.

Excellent functional outcomes at 6-month follow-up (mRS ≤ 1) were achieved by 91 patients (79.8%) within the ruptured aneurysm cohort. The logistic regression analysis did not reveal a significant association between homocysteine levels and excellent functional outcomes (OR 1.04; 95% CI 0.94-1.16).

## DISCUSSION

There was no significant discrepancy in homocysteine levels between patients with ruptured and those with unruptured intracranial aneurysms. Moreover, homocysteine did not demonstrate a correlation with either vasospasm or functional outcomes post-aneurysm rupture.

### Cardiovascular risk

A potent association exists between homocysteine and vascular dysfunction, and, consequently, cardiovascular risk. A panoply of molecular mechanisms have been implicated in homocysteine-mediated vascular damage, including oxidative stress, production of reactive oxygen species, thrombolysis impairment, mitochondrial modifications, and endothelial dysfunction. 1 13 14 15 16 Clinically, a 25% reduction in serum homocysteine levels has been linked with an 11% and 19% decrease in the risk of ischemic heart disease and stroke, respectively. 4 Furthermore, a study by Fan et al. (2017) even uncovered a significant dose-response surge in all-cause mortality tied to this molecule. 17

Stroke is also significantly associated with elevated homocysteine levels. 3 18 19 As such, clinical trials have been instigated to assess the benefits of supplementing folic acid, B6, and B12 in order to decrease homocysteine levels and potentially reduce stroke risk. However, the outcomes of these trials are far from consensus. 5 While some studies and meta-analyses, including the Heart Outcomes Prevention Evaluation (HOPE) 2, SU.FOL.OM3, and the China Stroke Prevention Trial (CSPPT), indicate a reduction in stroke risk, 5 20 21 22 others, such as the Vitamin Intervention for Stroke Prevention (VISP) 23 and the Norwegian Vitamin Trial (NORVIT), 24 show no such benefits or even suggest potential harm. Consequently, current guidelines do not routinely recommend vitamin supplementation.

### Intracranial aneurysm

Investigations exploring the relationship between homocysteine and intracranial aneurysms (IA) are scarce. Preclinical evidence suggests a methionine-rich diet may correlate with an elevated risk and expedited formation of IA, 25 and specific genetic variants of the homocysteine cycle have been linked with an increased IA risk. 26 27

Retrospective studies offer contradictory results. 28 29 Ren et al. (2017) 29 identified an association between homocysteine and IA patients [OR 2.2 (95%CI 1.19-4.06)], but both patients and controls exhibited exceptionally high homocysteine levels. As for subarachnoid hemorrhage (SAH), associations have been made with delayed cerebral ischemia, 10 disability, 30 and SAH itself. 31 Yet, some research contradicts these findings, 9 32 even demonstrating an inverse correlation between homocysteine levels and mortality after SAH. 8 Caution is urged when interpreting retrospective association studies due to potential publication bias and an inability to establish causality. 33 This study further fortifies the notion that homocysteine may not be associated with aneurysmal subarachnoid hemorrhage.

### Strengths and limitations

This study has limitations. Its retrospective nature, although apt for exploratory issues, inherently limits its capacity to provide robust causality information. The decision to analyze homocysteine in an ambulatory setting post-discharge was taken to control potential confounders associated with the acute inflammatory state, but this also introduces bias by excluding patients who did not survive their hospital stay.

Nonetheless, to the best of our knowledge, this is the inaugural study comparing patients with SAH to controls with unruptured aneurysms concerning these biomolecules. The relatively large sample size and adjusted analyses, which account for potential confounders, constitute key strengths of this study.

In conclusion, there were no differences regarding homocysteine serum levels between patients with ruptured and unruptured intracranial aneurysms. In patients with ruptured aneurysms homocysteine levels did not correlate to vasospasm or functional outcomes after discharge.
